# The Protection of Naturally Acquired Antibodies Against Subsequent SARS-CoV-2 Infection: A Systematic Review and Meta-Analysis

**DOI:** 10.1080/22221751.2022.2046446

**Published:** 2022-03-12

**Authors:** Qi Chen, Kongxin Zhu, Xiaohui Liu, Chunlan Zhuang, Xingcheng Huang, Yue Huang, Xingmei Yao, Jiali Quan, Hongyan Lin, Shoujie Huang, Yingying Su, Ting Wu, Jun Zhang, Ningshao Xia

**Affiliations:** a State Key Laboratory of Molecular Vaccinology and Molecular Diagnostics, National Institute of Diagnostics and Vaccine Development in Infectious Diseases, Strait Collaborative Innovation Center of Biomedicine and Pharmaceutics, School of Public Health, Xiamen University, Xiamen City, Fujian Province, People’s Republic of China; b The Research Unit of Frontier Technology of Structural Vaccinology of Chinese Academy of Medical Sciences, Xiamen City, Fujian Province, People's Republic of China

**Keywords:** SARS-CoV-2, COVID-19, naturally acquired antibody, reinfection, efficacy, meta-analysis

## Abstract

The specific antibodies induced by SARS-CoV-2 infection may provide protection against a subsequent infection. However, the efficacy and duration of protection provided by naturally acquired immunity against subsequent SARS-CoV-2 infection remain controversial. We systematically searched for the literature describing COVID-19 reinfection published before 07 February 2022. The outcomes were the pooled incidence rate ratio (IRR) for estimating the risk of subsequent infection. The Newcastle–Ottawa Scale (NOS) was used to assess the quality of the included studies. Statistical analyses were conducted using the R programming language 4.0.2. We identified 19 eligible studies including more than 3.5 million individuals without the history of COVID-19 vaccination. The efficacy of naturally acquired antibodies against reinfection was estimated at 84% (pooled IRR = 0.16, 95% CI: 0.14-0.18), with higher efficacy against symptomatic COVID-19 cases (pooled IRR = 0.09, 95% CI = 0.07-0.12) than asymptomatic infection (pooled IRR = 0.28, 95% CI = 0.14-0.54). In the subgroup analyses, the pooled IRRs of COVID-19 infection in health care workers (HCWs) and the general population were 0.22 (95% CI = 0.16-0.31) and 0.14 (95% CI = 0.12-0.17), respectively, with a significant difference (*P* = 0.02), and those in older (over 60 years) and younger (under 60 years) populations were 0.26 (95% CI = 0.15–0.48) and 0.16 (95% CI =  0.14-0.19), respectively. The risk of subsequent infection in the seropositive population appeared to increase slowly over time. In conclusion, naturally acquired antibodies against SARS-CoV-2 can significantly reduce the risk of subsequent infection, with a protection efficacy of 84%.

**Registration number:** CRD42021286222

## Introduction

The COVID-19 pandemic has caused devastating impacts on global health and the economy [1]. As of 15 February 2022, 412.3 million confirmed cases of COVID-19 and approximately 5.8 million deaths have been reported worldwide [2]. SARS-CoV-2 infection induces the production of detectable levels of specific antibodies in most patients within 3–6 weeks after infection, and only 2.0-8.5% of patients remained seronegative after 60 days post-infection [3]. Several studies reported that specific antibodies in patients with COVID-19 are continuously detected for at least 6–13 months after COVID-19 infection [4-6]. Previous studies indicated that naturally acquired antibodies after SARS-CoV-2 infection might protect against virus reinfection [7-9]. However, as these studies often included relatively small sample sizes with different follow-up times, populations and serology measurement methods, the actual extent to which primary infection protects against reinfection and how long protection lasts are still being debated.

As of 16 February 2022, 61.9% of the global population has received at least one dose of a COVID-19 vaccine. However, vaccine distribution is extremely uneven, with only 10.6% of people in low-income countries receiving at least one dose [10]. As the number of COVID-19 cases accumulates and vaccine campaigns continue, the extent and durability of the protective effect of natural immunity elicited by infection is critical to the pandemic prediction process, estimates of herd immunity and development of vaccination strategies [11-13]. Therefore, we conducted a systematic review and meta-analysis to evaluate the protective efficacy of naturally acquired humoral immunity against subsequent SARS-CoV-2 infection in unvaccinated individuals.

## Methods

### Search strategy

We systematically searched for the relevant literature published before 07 February 2022 in six databases, including three peer-reviewed databases (PubMed, Embase, and Web of Science) and three preprint platforms (medRxiv, bioRxiv, and Europe PMC). Key search terms included the following: SARS-CoV-2, immunity, protection, reinfection and nucleic acid testing. The full search strategy was described in Supplementary Table 1. We also conducted a secondary reference search on all eligible studies and nine relevant review articles [11,14-21]. The protocol of this systematic review and meta-analysis has been registered at PROSPERO (Registration number: CRD42021286222) [22].

### Selection criteria

Two investigators (“QC” and “KXZ”) independently assessed all retrieved publications. Discrepancies were resolved by discussion with a third investigator (“XHL”). Eligible studies must meet all of the following criteria: (1) longitudinal study in population without a history of COVID-19 vaccination, (2) SARS-CoV-2 serology testing at baseline to discriminate the previously infected and uninfected populations, (3) confirmation of COVID-19 cases by nucleic acid testing during follow-up, (4) the study must have compared the risk of SARS-CoV-2 reinfection/infection between baseline seropositive and seronegative groups, and (5) a sample size of >10 participants in each group. We excluded studies that met any of the following criteria: (1) the study used odds ratio as an effect size indicator and did not report original data, and (2) the baseline seronegative group included subjects with a known COVID-19 history.

### Data extraction and quality assessment

Two investigators (“KXZ” and “XCH”) extracted the following data using a standardized electronic data collection form: study title, publication or preprint date, journal or preprint platform, authors, study location, demographic characteristics of the study population, follow up time, the method of the serology measurement, sample sizes and reinfection/infection cases in baseline seropositive or seronegative groups, the definition of reinfection, whether researchers attempted to adjust for any potential covariates, IRR and 95% confidence interval (95% CI). If the IRR was not able to be obtained from the study, a 2×2 contingency table was constructed to calculate it. When no cases of reinfection/infection were detected in the baseline seronegative or seropositive groups during follow-up, “0.05” was used to evaluate an estimate of the relative risk. The quality of the included studies was independently evaluated using the Newcastle–Ottawa Scale (NOS) by two investigators (“KXZ” and “XCH”). The NOS contains three categories (8 subcategories), and a maximum of 9 stars can be allotted to each study. A score of 0–3 stars was considered a low-quality study, a score of 4–6 stars was considered a moderate-quality study, and a score of 7–9 stars was considered a high-quality study. Extracted data and quality assessment scores were checked by a third investigator (“XHL”), and disagreements were resolved through discussion.

### Statistical analysis

The primary outcome was the risk of SARS-CoV-2 reinfection/infection between the baseline seropositive and seronegative groups. The second outcome was the risk of symptomatic and asymptomatic COVID-19 between the two groups. The subjects with a positive PCR test for SARS-CoV-2 after the baseline seropositive result were defined as reinfection cases. Heterogeneity was assessed using the Q and *I*² statistics, with *P *< 0.05 and *I²*>50% indicating significant heterogeneity. A suitable model was used to generate pooled IRR and 95% CIs. Subgroup analyses of the primary outcome were performed in the following groups: peer-review status (peer-review or preprint), the type of target antibodies (S protein or N protein), population (HCWs or general population), age (< 60 years old or ≥ 60 years old), whether studies reported adjusted results (adjusted or unadjusted), the method for monitoring infection cases (passive monitoring or active monitoring), and the definition of reinfection (strict definition or loose definition). The classification criteria for each subgroup are described in the Supplementary Table 2. Bubble plots were used to explore the changing trends of the protection provided by naturally acquired antibodies. Funnel plots (log risk ratio against standard errors) and Begg’s test were used to examine the potential for publication bias. The one-study-at-a-time method (OAT) was used to assess the reliability of the results in the sensitivity analysis.

Statistical analyses were conducted using meta libraries in the R programming language 4.0.2 [23]. A two-sided *P value* < 0.05 was considered statistically significant.

## Results

**Figure 1.**
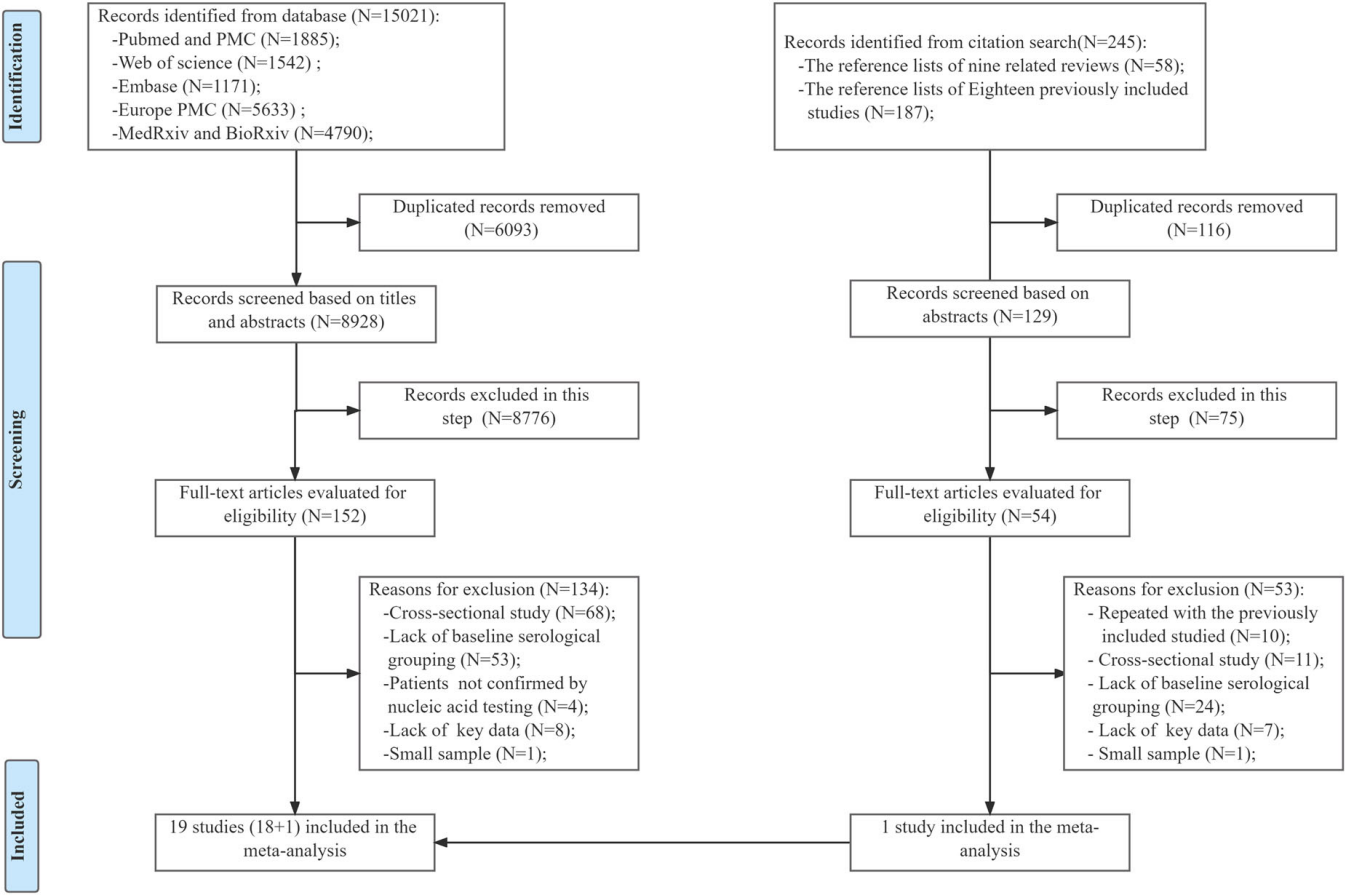
Flow diagram of the included studies. One study identified from the second citation search was included in the meta-analysis. [24].

A total of 15021 relevant records were identified, of which 6093 duplicate records were removed. According to the titles and abstracts, 8776 irrelevant records were excluded, and the full texts of 152 articles were assessed. Additionally, 245 relevant records were identified from the second citation search. Finally, 19 studies were eligible and included in this meta-analysis (Figure 1).

The 19 eligible studies included more than 3.5 million COVID-19 unvaccinated individuals without the history of COVID-19 vaccination. The sample sizes of the included studies ranged from 209 to 3257478 (median: 3249, IQR: 653-10582). Eight studies were conducted in the UK, four in the USA, three in Switzerland, one in Italy, one in Qatar, one in Nicaragua, and one in Sweden. The mean/median ages of the enrolled participants in fifteen studies (15/19) were less than 60 years old, those in another two studies (2/19) were more than 60 years old, and the remaining two studies (2/19) separately analyzed both the two age groups. The study populations mainly included HCWs, the general population, residents of a care home, marine recruits and hemodialysis patients. Most studies initiated between January 2020 and June 2020, and only one study started in October 2020 [24]. The length of the follow-up time ranged from 4.0–13.0 months [24,25]. Two main methods, enzyme-linked immunosorbent assay (ELISA) and chemiluminesent micropaticle immunoassay (CMIA), were used to measure titers of blinding antibodies against SARS-CoV-2, and no studies grouped participants according to the baseline status of neutralizing antibodies. As most studies started during the early stage of the COVID-19 pandemic, the persistency of SARS-CoV-2 RNA has not been clearly understood, so varied window periods between the positive PCR test and the baseline seropositive or previous positive RNA result were adopted in defining reinfection in different studies. The main characteristics of all included studies are summarized in Table 1.

**Table 1.** Description of included studies and reported outcomes.

NO.	Authors Years	Population and Location	Age (years)	Study Time	Length of Follow-up, mo^c^	The Definition of reinfection	Serologic Assay	Effect Measure	Adjusted Variables	Quality assessment^e^
1	John T. Wilkins MD et al, 2020 [44]	6510 HCWs in Chicago, USA	Mean (SD) 41(12)	2020.05.26-2021.01.08	7.5	A positive PCR result detected more than 90 days after baseline seropositive result, or the first positive PCR test plus 1 or more of the following characteristics: in-home exposure to someone infected with SARS-CoV-2, consistent symptoms, or a physician diagnosis of active infection.	CMIA; Anti-NP IgG tested by ARCHITECT Immunoassay System (Abbott)	Adjusted RR	Age, Gender, Race and Occupation	7 (HQ)
2	Sheila F Lumley et al, 2020 [45]	About 12600 HCWs in Oxford shire, UK	Median (IQR) 38 (29,49)	2020.04.23-2020.11.30	7.3	A positive PCR result detected more than 60 days after the previous PCR positive test.	1. ELISA; Anti-S IgG tested by an ELISA platform developed by the University of Oxford 2. CMIA; Anti-NP IgG tested by ARCHITECT Immunoassay System (Abbott)	Adjusted RR	Calendar time, Age, and Gender,	6 (MQ)
3	H. Abo-Leyah et al, 2021 [46]	2063 HCWs in Scotland, UK	Median 46	2020.05.28-2020.12.02	6.2	A positive PCR result detected more than 60 days after a baseline seropositive result.	CLIA; Anti-S total antibodies tested by the Siemens SARS-CoV-2 total antibody assay	Unadjusted HR	Age and Gender	6 (MQ)
4	Anna Jeffery-Smith et al, 2021 [47]	103 care homes residents and 106 staffs in London, UK	Median (IQR) A care home residents: 84 (76-89) L care home residents: 85 (78-89)	2020.05-2020.10	5.0	Reinfection was defined as an individual testing positive for the SARS-CoV-2 RT–PCR test while having evidence of previous seropositivity detected using any assay (more than 90 days ago).	1.ELISA; Anti-RBD IgG and anti-S IgG tested by an uncommercially kit 2.CMIA; Anti-NP IgG tested by a commercial assay (Abbott, Illinois, United States)	Unadjusted RR^b^	-	5 (MQ)
5	Raymond A. Harvey et al, 2021^d^[48]	3257478 general population in USA	Median (SD) 48 (20)	2020.01.08-2020.08.26	7.6	A positive PCR result detected more than 90 days after the baseline seropositive result.	–	Unadjusted RR	–	6 (MQ)
6	Adrian M. Shields et al, 2021^a^ [49]	1507 HCWs in the West Midlands region, UK	Median (IQR) 37 (29-47)	2020.05-2021.01	8.0	A positive PCR result detected after a baseline seropositive result.	ELISA; Anti-S total antibodies tested by a commercially total antibody assay (Product code: MK654, Birmingham)	Adjusted RR	Age, Gender, Ethnicity and Smoking	7 (HQ)
7	Antonio Leidi et al (1), 2021 [50]	8344 general population in Geneva, Switzerland	Mean (SD) Pos: 47 (17) Neg: 47 (16)	2020.04.03 -2021.01.25	9.8	Two independent adjudicators evaluated suspected cases based on the reason for testing, subject’s illness history (including date of symptom onset), and the value and temporal evolution in RT–PCR cycle threshold (Ct).	ELISA; Anti-S IgG tested by a commercially kit (Euroimmun, Lübeck, Germany #EI 2606–9601 G)	Unadjusted HR	–	7 (HQ)
8	Candice L. Clarke et al, 2021 [51]	356 hemodialysis patients in London, UK	Mean (IQR) Pos: 65 (55-73) Neg: 68 (55-77)	2020.02.24-2021.01.01	10.3	A positive PCR result detected more than 60 days after a baseline seropositive result.	1. ELISA; Anti-RBD IgG tested by an uncommercially kit (Imperial SARS-CoV-2 Hybrid DABA) 2.CMIA; Anti-NP IgG tested by ARCHITECT Immunoassay System (Abbott)	Unadjusted RR^b^	–	6 (MQ)
9	Victoria Jane Hall et al, 2021 [52]	25661 HCWs in UK	Median (IQR) 46 (35-54)	2020.02.01-2021.01.11	14.3	A positive PCR result detected more than 90 days after a previous positive PCR test, or at least 4 weeks after a baseline seropositive result.	–	Unadjusted RR	Week group, Age group, Gender, Ethnicity, Staff role, Index of multiple deprivation, Region	8 (HQ)
10	Andrew G Letizia et al, 2021 [53]	3249 marine recruits in Parris Island, USA	Range 18–20	2020.05.11-2020.11.02	5.8	A positive PCR result detected more than 14 days after a baseline seropositive result.	ELISA; Anti-RBD IgG and anti-S IgG tested by an uncommercially kit	Adjusted HR	Age, Gender and Race	7 (HQ)
11	Mattia Manica et al, 2021 [27]	6074 general population in the Autonomous Province of Trento, Italy	Median (IQR) 50 (32-63)	2020.05.05-2021.01.31	8.9	A positive PCR result detected after a baseline seropositive result.	CMIA; Anti-NP IgG tested by ARCHITECT Immunoassay System (Abbott)	Adjusted RR	Age	8 (HQ)
12	L. J. Abu-Raddad et al, 2021^d^ [54]	43044 seropositive population in Qatar and 149934 seronegative population in 167 nationalities	Median (IQR) POS: Male 38 (31-47), Female is 35 (28-45) Neg: Male 39 (30-50), Female 35 (28-47)	2020.04.16-2020.12.31	8.5	A PCR-positive swab at least 14 days after the first positive antibody test and no PCR-positive swab within the 45 days preceding the reinfection swab.	ECLIA; Anti-NP IgG tested by Roche Elecsys® Anti-SARS-CoV-2 assay (Roche, Switzerland)	Unadjusted RR	–	6 (MQ)
13	Maria Krutikov et al, 2021 [24]	682 residents and 1429 staff members of long-term care facilities in UK	Median (IQR) Residents 86 (79–91) Staff members 47 (34–56)	2020.10.01-2021.02.01	4.0	A positive PCR result detected more than 28 days after the initial seropositive result.	CMIA; Anti-NP IgG tested by ARCHITECT Immunoassay System (Abbott)	Unadjusted RR^b^	–	5 (MQ)
14	Antonio Leidi et al (2), 2021 [55]	10582 essential workers in Geneva, Switzerland	Mean (SD) Pos: 44 (11) Neg: 45 (11)	2020.05-2021.01	8.0	Positive RT–PCR or antigenic rapid diagnostic test in seropositive individuals were clinically investigated by two independent adjudicators.	ELISA; Anti-S IgG tested by a commercially kit (Euroimmun anti-SARS-CoV-2 IgG ELISA)	Adjusted HR	Age, Sex, Smoking status, Obesity and Formal educational level	7 (HQ)
15	Hannah E Maier et al, 2021 [25]	2338 general population in Managua, Nicaragua	Mean (SD) 24 (18)	2020.03.01-2021.03.31	13.0	A positive PCR result detected after a baseline seropositive result.	ELISA; Anti-RBD IgG tested by an uncommercially kit following the Krammer laboratory protocol	Unadjusted RR^b^	–	8 (HQ)
16	Sebastian Havervall et al, 2021 [56]	300 HCWs in Stockholm, Sweden	Median (IQR) 46 (35-54)	2020.04.09-2021.02.26	10.6	A positive PCR result detected more than 7 months after a baseline seropositive result.	Luminex; Anti-S IgG tested by a bead-based high-throughput multiplex assay based on the FlexMap3D (Luminex Corp.) platform	Unadjusted RR^b^	–	5 (MQ)
17	Charles F Schuler 4^th^ et al, 2021 [57]	653 subjects were either HCWs or patients with a high risk of exposure to COVID-19 in Michigan, USA	Median (IQR) 41 (31-51)	2020.05-2021.02	9.0	A positive PCR result detected after a baseline seropositive result.	1. ECLIA; Anti-NP total antibodies tested by Roche Elecsys® Anti-SARS-CoV-2 assay (Roche, Switzerland) 2.CLIA; Anti-S total antibodies tested by an ADVIA Centaur XPT analyzer (Siemens)	Unadjusted RR^b^	–	5 (MQ)
18	Philipp Kohler et al, 2021 [58]	4812 HCWs in Northern and Eastern Switzerland	Median38.9	2020.06.22-2021.03.09	8.5	A positive PCR result detected more than 90 days after a baseline seropositive result.	ECLIA; Anti-NP total antibodies (Roche, Switzerland)	Unadjusted RR	–	5 (MQ)
19	Luke Muir et al, 2021 [59]	217 patients in blood and transplant waiting list for renal transplantation in UK	Mean (SD) Pos: 54.5 (11.9) Neg: 53.6 (12.7)	2020.06-2021.01	7.0	A positive PCR result detected more than 60 days after a baseline seropositive result.	ELISA; Anti-S IgG and anti-N IgG tested by uncommercially kits	Unadjusted RR^b^	–	7 (HQ)

HCWs: Health-care Workers; SD: Standard Deviation; IQR: Interquartile Range; CMIA: Chemiluminescent Microparticle Immunoassay; CLIA: Chemiluminescence Immunoassay; ELISA: Enzyme-Linked Immunosorbent Assay; RR: Relative Risk; HR: Hazard Ratio; HQ: High Quality; MQ: Medium Quality; LQ: Low Quality;

^a^ This article is a preprint and has not been peer-reviewed.

^b^ A 2×2 contingency table was constructed to calculate it, as the effect size is not available in the article.

^c^ Length of Follow-up (months) = [(the date of study start – the date of study end)÷365×12], or (the month of study start – the month of study end).

^d^ This is a database study.

^e^ Quality assessment of the included studies using the NOS

The two independent investigators evaluated study quality according to the NOS. The consistency of the evaluated NOS items was 95.4% (147/152), which suggested that interrater reliability was high (Supplementary Table 3). The scores for study quality ranged from 5 to 8. Nine studies were determined to be with high quality, ten studies with moderate quality, and no study was judged as with low quality (Supplementary Table 4).

### The protection of naturally acquired antibodies against SARS-CoV-2 reinfection

**Figure 2.**
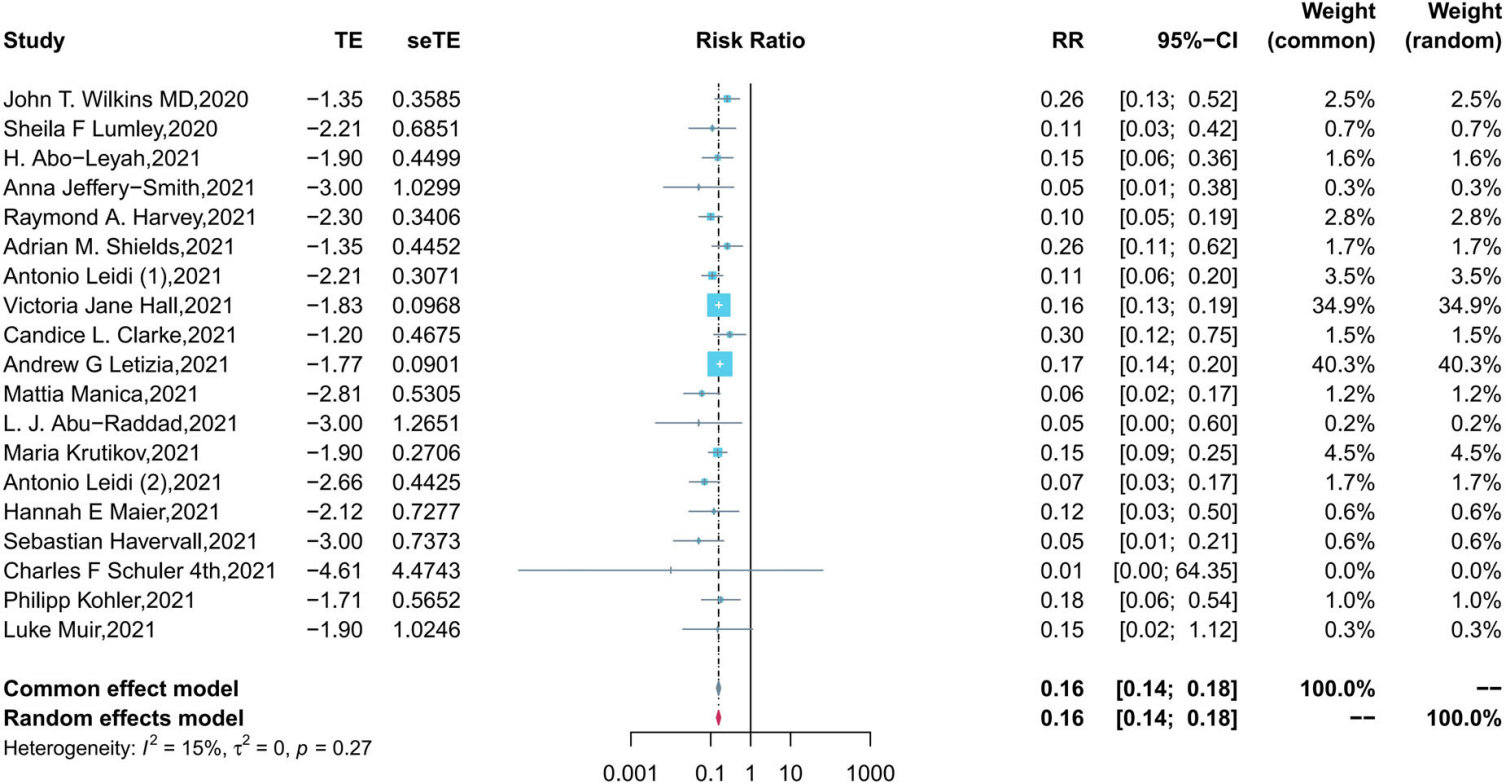
Forest plot of the pooled incidence rate ratio for SARS-CoV-2 infection comparing baseline seropositive and seronegative individuals.

Figure 2 presents the pooled results for the protection of naturally acquired antibodies against future SARS-CoV-2 infection. In fixed effect meta-analysis models (*I^2^*^ ^= 15%, *P *= 0.27), we observed significant protection against SARS-CoV-2 reinfection in the seropositive population compared with seronegative individuals (pooled IRR = 0.16, 95% CI = 0.14-0.18). In the sensitivity analysis, the pooled IRRs of remaining studies ranges from 0.14–0.16 after removing any one of the studies, which suggested the good reliability of the pooled IRR (Supplementary Figure 1). Except for one extreme value, the funnel plot indicated that most of the included studies were symmetrically distributed in the triangular area (Supplementary Figure 2). Begg’s test similarly suggested no publication bias in the included studies (*P* = 0.22).

In the subgroup analyses, the pooled IRRs in HCWs and the general population were 0.22 (95% CI = 0.16–0.31, *I^2^*  = 19%, *P* = 0.28) and 0.14 (95% CI = 0.12–0.17, *I^2^* = 41%, *P* = 0.13), respectively, with a significant difference (*P* = 0.02) observed between the two populations (Supplementary Figure 3). The incidence risk ratio of reinfection was lower in participants aged less than 60 years than in participants aged greater than 60 years (0.16, 95% CI =  0.14-0.19 *vs* 0.26, 95% CI = 0.15–0.48), however, differences (*P* = 0.12) between the two age groups were not significant (Supplementary Figure 4). In addition, no significant differences (all *P *> 0.05) were observed in the subgroup analysis of the peer review status of articles, the types of target antibodies, the definition of reinfection cases, and whether studies reported adjusted results (Supplementary Figures 5-9).

**Figure 3.**
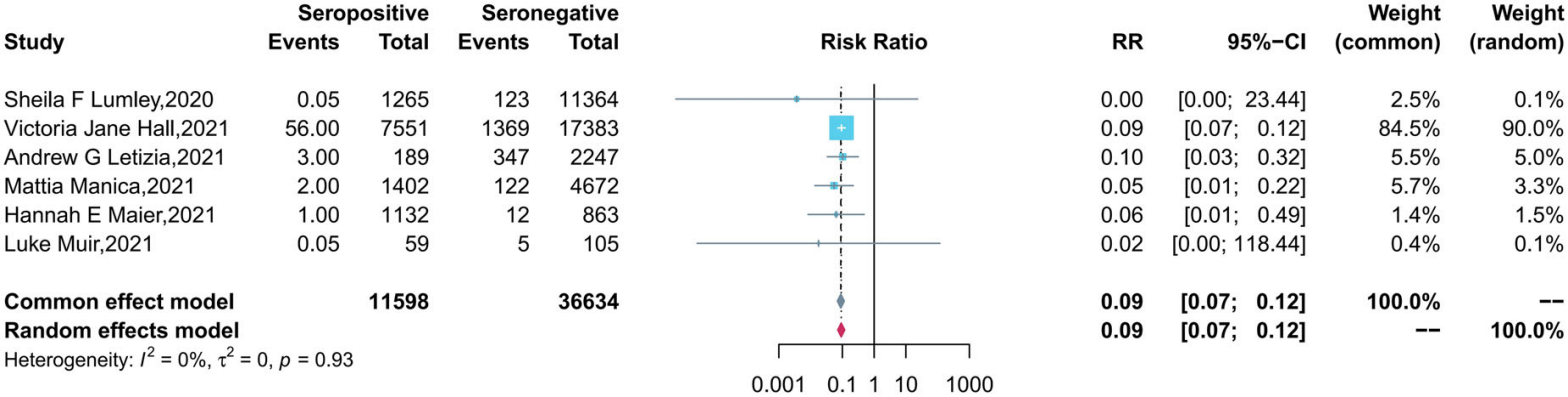
Forest plot of the protection provided by naturally acquired antibodies against future symptomatic COVID-19 between baseline seropositive and seronegative individuals.

**Figure 4.**
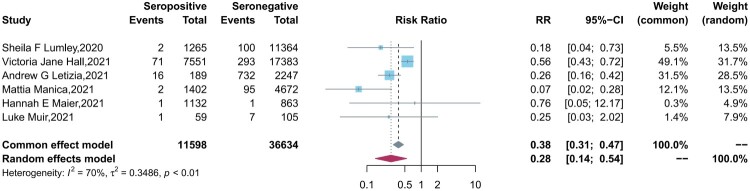
Forest plot of the protection provided by naturally acquired antibodies against future asymptomatic COVID-19 between baseline seropositive and seronegative individuals.

Six studies reported the protection of the antibodies induced by a previous infection against future symptomatic and asymptomatic reinfections between baseline seropositive and seronegative groups. Similar to the results of vaccine effectiveness, natural infections provided a lower level of protection against asymptomatic infection (pooled IRR = 0.28, 95% CI = 0.14-0.54) than symptomatic COVID-19 cases (pooled IRR = 0.09, 95% CI = 0.07-0.12) (Figures 3–4).

Ten studies that reported the mean/median follow-up times were included in the bubble plot to explore the changing trends of the protection provided by naturally acquired antibodies after a prior COVID-19 infection, the protection appeared to decrease slowly over time (Supplementary Figure 10).

## Discussion

This systematic review and meta-analysis, including 19 studies and > 3.5 million individuals without the history of COVID-19 vaccination, provided a synthesis of the evidence that naturally acquired antibodies against SARS-CoV-2 significantly reduce the risk of subsequent infection. The efficacy of natural infection with detectable antibodies against reinfection was estimated at 84%, with higher efficacy against symptomatic COVID-19 cases than asymptomatic infection. Natural humoral immunity seems to provide similar or greater protection than COVID-19 vaccines in the ensuing thirteen months after a prior infection [25,26].

However, it is noted that there was high heterogeneity (*I²* = 70%, *P*<0.01) among the asymptomatic reinfection studies. After further subgroup analyses, we found the too stringent definition of asymptomatic cases in one study with large sample size (enrolled 25661 HCWs), which excluded PCR positive subjects who were pauci-symptomatic from asymptomatic cases, lead to the low protection efficacy of natural immunity in this study [27]. However, although excluding this study decreases the heterogeneity (*I²* = 0%, *P *= 0.42), it does not change the conclusion a lot (pooled IRRs = 0.21 *vs* 0.28), which indicates the reliability of the result.

In the subgroup analyses, we found that the protection of antibodies in HCWs was lower than that of the general population (pooled IRRs of 0.22 *vs* 0.14). Many studies have found that HCW populations have a higher seropositive rate and infection risk than the general population [28-30]. High-risk medical procedures with repeated and close contact with patients significantly increased the frequency and intensity of exposure for HCWs, which may directly affect the protective effect of antibodies. Furthermore, HCW populations usually have a high frequency of COVID-19 nucleic acid testing, which may lead HCWs with asymptomatic COVID-19 to be more likely to be identified [31]. This increase in testing is another possible cause of the low level of protection of antibodies against SARS-CoV-2 that was observed in the HCW population.

We also observed relatively lower protection against reinfection in the seropositive population aged greater than 60 years compared with younger individuals. A large amount of strong evidences suggested that the reductions in the antibody response induced by vaccination are significantly associated with increasing age [32,33]. However, the dynamic change in antibodies acquired from natural infection differs from that induced by vaccinations. Antibodies were detected at higher levels in the population over 60 years of age in the early recovery period than in the younger population but then decayed at a faster rate [34,35]. This difference may be correlated with the negative alterations in the immune system with increasing age, which is known as immunosenescence [36]. The differences in the dynamics of antibody responses may be a cause of the lower level of protection in the population aged greater than 60 years. These results suggest that even with a history of SARS-CoV-2 infeciton, elderly individuals and HCWs need to be vaccinated in a timely manner to obtain stronger protection against future infection.

Notably, most studies included in this systematic review were conducted between January 2020 and March 2021, indicating that the majority of the subjects included in those studies had not experienced the outbreaks of Delta, Omicron and other new variants during the pandemic. According to data from the Global Initiative on Sharing All Influenza Data (GISAID), as of 6 February 2022, Omicron (approximately 92%) and Delta (approximately 7%) were the dominant strains in the COVID-19 pandemic [37]. Substantial mutations of the spike and nucleocapsid proteins lead to higher viral loads and increased transmission and adversely affect the protection provided by antibodies acquired from prior infection [38,39]. A retrospective study in South Africa indicated that a higher risk of reinfection was observed for Omicron than for Beta and Delta, suggesting that Omicron has a stronger immune escape capacity [40,41]. The herd immunity constructed from COVID-19 vaccination and natural infection may become a remote goal. Further research is needed to determine the level of protection provided naturally acquired antibodies against mutant strains, as well as the persistence of protection.

This study was also subject to other limitations. Firstly, the requested window periods between the positive RNA test and the baseline seropositive or previous positive RNA result in the definitions of reinfection were inconsistent in the included studies. A looser definition of reinfection in some studies may lead to some patients with prolonged viral shedding being misunderstood as reinfection cases and underestimating the protection of antibodies against SARS-CoV-2 [16,42]. However, in the subgroup analysis of studies with strict or loose definition, the protection efficacy were similar in the two subgroups which implies the negligible impact of this factor. Secondly, adaptive immunity activated by SARS-CoV-2 infection, including B cells, CD4+ T cells, and CD8+ T cells, provides long-term protection against reinfection [5,43]. Some hidden COVID-19 infectors who were seronegative but positive for cellular immunity were included in the baseline seronegative group, which may have a negative effect on the estimates of protection efficacy.

In conclusion, antibodies acquired from prior infection can provide strong protection against reinfection with an efficacy of 84%. Meanwhile, the protection provided by naturally acquired antibodies in HCWs and elderly individuals aged greater than 60 years was relatively low compared with the general population and younger population. During this evolving pandemic, the protection of naturally acquired antibodies against mutant strains, as well as the persistence of protection, requires further study.

## Supplementary Material

Supplemental Material

## Data Availability

Data extracted from included studies can be obtained from https://github.com/chenqi160/Data.
