# Supplementary material for: The Protection of Naturally Acquired Antibodies Against Subsequent SARS-CoV-2 Infection: A Systematic Review and Meta-Analysis
Source: Emerg Microbes Infect. 2022 Mar 12;11(1):793–803. doi: 10.1080/22221751.2022.2046446 (PMC8920404; doi:10.1080/22221751.2022.2046446)

**Supplementary File**

**The Protection of Naturally Acquired Antibodies Against Subsequent SARS-CoV-2 infection: A Systematic Review and Meta-Analysis**

**Contents**

[Supplementary Table 1. Search strategy for peer-reviewed databases and preprint platforms 3](#_Toc92228945)

[Supplementary Table 2: The classification criteria for subgroup 5](#_Toc92228946)

[Supplementary Table 3. The consistency of Newcastle–Ottawa scale scores between two independent investigators 6](#_Toc92228947)

[Supplementary Table 4. Quality assessment of the included studies using the NOS* 8](#_Toc92228948)

[Supplementary Figure 1. Sensitivity analysis for pooled incidence rate ratio 10](#_Toc92228949)

[Supplementary Figure 2. Funnel plot for publication bias 11](#_Toc92228950)

[Supplementary Figure 3. Forest plot of the pooled incidence rate ratio for SARS-CoV-2 infection comparing baseline seropositive with seronegative individuals in HCWs subgroup and general population subgroup 12](#_Toc92228951)

[Supplementary Figure 4. Forest plot of the pooled incidence rate ratio for SARS-CoV-2 infection comparing baseline seropositive with seronegative individuals in <60 years old subgroup and ≥60 years old subgroup 13](#_Toc92228952)

[Supplementary Figure 5. Forest plot of the pooled incidence rate ratio for SARS-CoV-2 infection comparing baseline seropositive with seronegative individuals in peer-review subgroup and preprint subgroup 14](#_Toc92228953)

[Supplementary Figure 6. Forest plot of the pooled incidence rate ratio for SARS-CoV-2 infection comparing baseline seropositive with seronegative individuals in S protein subgroup and N protein subgroup 15](#_Toc92228954)

[Supplementary Figure 7. Forest plot of the pooled incidence rate ratio for SARS-CoV-2 infection comparing baseline seropositive with seronegative individuals in adjusted subgroup and unadjusted subgroup 16](#_Toc92228955)

[Supplementary Figure 8. Forest plot of the pooled incidence rate ratio for SARS-CoV-2 infection comparing baseline seropositive with seronegative individuals in passive monitoring subgroup and active monitoring subgroup 17](#_Toc92228956)

[Supplementary Figure 9. Forest plot of the pooled incidence rate ratio for SARS-CoV-2 infection comparing baseline seropositive with seronegative individuals in strict definition subgroup and loose definition subgroup 18](#_Toc92228957)

[Supplementary Figure 10. The changing trend of incidence rate ratio after prior infection 19](#_Toc92228958)

# Supplementary Table 1. Search strategy for peer-reviewed databases and preprint platforms

| **Database** | **Step** | **Search strategy** |
| --- | --- | --- |
| PubMed  (PMC) | #1 | (((((COVID-19[Title/Abstract]) OR (Coronavirus disease 2019[Title/Abstract])) OR (2019-nCoV[Title/Abstract])) OR (severe acute respiratory syndrome coronavirus 2[Title/Abstract])) OR (SARS-CoV-2[Title/Abstract])) |
|  | #2 | ((((((Seropositive[Title/Abstract]) OR (Seronegative[Title/Abstract])) OR (Antibody[Title/Abstract])) OR (immunity[Title/Abstract])) OR (immunological[Title/Abstract])) OR (Antibodies[Title/Abstract])) |
|  | #3 | (((((((((((Risk[Title/Abstract]) OR (protect[Title/Abstract])) OR (protection[Title/Abstract])) OR (protective[Title/Abstract])) OR (protected[Title/Abstract])) OR (prevention[Title/Abstract])) OR (Preventive[Title/Abstract])) OR (effect[Title/Abstract])) OR (effective[Title/Abstract])) OR (Effectiveness[Title/Abstract])) OR (efficacy[Title/Abstract])) |
|  | #4 | (((infection[Title/Abstract]) OR (infected[Title/Abstract])) OR (reinfection[Title/Abstract])) |
|  | #5 | (((((PCR[Title/Abstract]) OR (RT-PCR[Title/Abstract])) OR (Polymerase Chain Reaction[Title/Abstract])) OR (Real-Time Polymerase Chain Reaction[Title/Abstract])) OR (sequencing[Title/Abstract])) OR (Nucleic Acid[Title/Abstract])) |
|  | #6 | #1 AND #2 AND #3 AND #4 AND #5 |
| Web of science | #1 | COVID-19 (Title) or COVID-19 (Abstract) or Coronavirus disease 2019 (Title) or Coronavirus disease 2019 (Abstract) or 2019-nCoV (Title) or 2019-nCoV (Abstract) or severe acute respiratory syndrome coronavirus 2 (Title) or severe acute respiratory syndrome coronavirus 2 (Abstract) or SARS-CoV-2 (Title) or SARS-CoV-2 (Abstract) |
|  | #2 | Seropositive (Title) or Seropositive (Abstract) or Seronegative (Title) or Seronegative (Abstract) or Antibody (Title) or Antibody (Abstract) or immunity (Title) or immunity (Abstract) or immunological (Title) or immunological (Abstract) or Antibodies (Title) or Antibodies (Abstract) |
|  | #3 | Risk (Title) or Risk (Abstract) or protect (Title) or protect (Abstract) or protection (Title) or protection (Abstract) or protective (Title) or protective (Abstract) or protected (Title) or protected (Abstract) or prevention (Title) or prevention (Abstract) or Preventive (Title) or Preventive (Abstract) or effect (Title) or effect (Abstract) or effective (Title) or effective (Abstract) or Effectiveness (Title) or Effectiveness (Abstract) or efficacy (Title) or efficacy (Abstract) |
|  | #4 | infection (Title) or infection (Abstract) or infected (Title) or infected (Abstract) or reinfection (Title) or reinfection (Abstract) |
|  | #5 | PCR (Title) or PCR (Abstract) or RT-PCR (Title) or RT-PCR (Abstract) or Polymerase Chain Reaction (Title) or Polymerase Chain Reaction (Abstract) or Real-Time Polymerase Chain Reaction (Title) or Real-Time Polymerase Chain Reaction (Abstract) or sequencing (Title) or sequencing (Abstract) or Nucleic Acid (Title) or Nucleic Acid (Abstract) |
|  | #6 | #1 AND #2 AND #3 AND #4 AND #5 |
| Embase | #1 | 'covid 19':ab,ti OR 'coronavirus disease 2019':ab,ti OR '2019 ncov':ab,ti OR 'severe acute respiratory syndrome coronavirus 2':ab,ti OR 'sars cov 2':ab,ti |
|  | #2 | seropositive:ab,ti OR seronegative:ab,ti OR antibody:ab,ti OR immunity:ab,ti OR immunological:ab,ti OR antibodies:ab,ti |
|  | #3 | risk:ab,ti OR protect:ab,ti OR protection:ab,ti OR protective:ab,ti OR protected:ab,ti OR prevention:ab,ti OR preventive:ab,ti OR effect:ab,ti OR effective:ab,ti OR effectiveness:ab,ti OR efficacy:ab,ti |
|  | #4 | infection:ab,ti OR infected:ab,ti OR reinfection:ab,ti |
|  | #5 | pcr:ab,ti OR 'rt pcr':ab,ti OR 'polymerase chain reaction':ab,ti OR 'real-time polymerase chain reaction':ab,ti OR sequencing:ab,ti OR 'nucleic acid':ab,ti |
|  | #6 | #1 AND #2 AND #3 AND #4 AND #5 |
| Europe PMC | #1 | ("COVID-19" OR "SARS-CoV-2") |
|  | #2 | ("Sero*" OR "Antibody" OR "immun*" OR "Antibodies") |
|  | #3 | ("Risk" OR "protect*" OR "effect*") |
|  | #4 | ("infection" OR "infected" OR "reinfection") |
|  | #5 | ("PCR" OR "RT-PCR" OR "Polymerase Chain Reaction" OR "Real-Time Polymerase Chain Reaction") |
|  | #6 | Type: Preprints |
|  | #7 | #1 AND #2 AND #3 AND #4 AND #5 AND #6 |
| medRxiv, bioRxiv | #1 | (COVID-19 or SARS-CoV-2) AND (Sero* OR Antibod*) AND (INFECT* OR reinfect*) and (protect*) and (PCR) |

* We systematically searched for the relevant literatures published before 07 February 2022 in above databases.

# Supplementary Table 2: The classification criteria for subgroup

| **Subgroup** | **Classification criteria** |
| --- | --- |
| **Peer review status** |  |
| Peer review | The study was published in a peer-reviewed journal. |
| Preprint | The study was submitted to a preprint platform. |
| **Target antibodies** |  |
| S protein | The anti-S antibody was measured in this study. |
| N protein | The anti-N antibody was measured in this study. |
| **Population** |  |
| HCWs | Health care workers include physicians, nurses, emergency medical personnel, dental professionals and students, medical and nursing students, laboratory technicians, pharmacists, hospital volunteers, and hospital administrative staff.  (https://www.cdc.gov/vaccines/adults/rec-vac/hcw.html) |
| General population | The studies included the community population and did not target a specific population. |
| **Age** |  |
| < 60 years old | The median or mean age of participants was < 60 years. |
| ≥ 60 years old | The median or mean age of participants was ≥ 60 years. |
| **Adjusted** |  |
| Yes | The study adjusted for some potential confounders. |
| No | The study did not adjust for any potential confounders. |
| **Monitoring method** |  |
| Passive monitoring | The results of nucleic acid testing were sourced from the electronic record or the self-report of participants. |
| Active monitoring | Participants regularly received nucleic acid testing. |
| **Definition of “Reinfection”** |  |
| Strict | In the baseline seropositive cohort: 1) Person with a positive SARS-CoV-2 RNA test at more than 90 days since the previous SARS-CoV-2 infection was defined as a reinfection case. 2) Person with a positive SARS-CoV-2 RNA test at more than 90 days after baseline seropositivity was defined as reinfection case. 3) Some independent adjudicators evaluated suspected cases based on multiple factors, such as the reason for testing, subject’s illness history, the value and temporal evolution in RT–PCR cycle threshold (Ct). |
| Loose | Person with a positive SARS-CoV-2 RNA test after baseline seropositivity was defined as a reinfection case. There were no limitation for the window period between the positive SARS-CoV-2 RNA test and the baseline seropositive or previous positive RNA result. These studies may have the risk that patients diagnosed with COVID-19 presenting with prolonged viral shedding were inappropriately defined as reinfection cases and the protection of antibodies against SARS-CoV-2 was underestimated. |

# Supplementary Table 3. The consistency of Newcastle–Ottawa scale scores between two independent investigators

| NO. | Study | Selection | | | | | | | |  | Comparability | |  |  |  | | Outcome | |  |
| --- | --- | --- | --- | --- | --- | --- | --- | --- | --- | --- | --- | --- | --- | --- | --- | --- | --- | --- | --- |
|  |  | Representativeness of the exposed cohort | | Selection of the non-exposed cohort | | Ascertainment of exposure | | Demonstration that outcome of interest was not present at start of study | |  | Comparability of cohorts on the basis of the design or analysis^^^ | |  | Assessment of outcome | | Was follow up long enough for outcomes to occur | | Adequacy of follow up of cohorts | |
|  |  | I1 | I2 | I1 | I2 | I1 | I2 | I1 | I2 |  | I1 | I2 |  | I1 | I2 | I1 | I2 | I1 | I2 |
| 1 | John T. Wilkins MD  2020 | N | N | Y | Y | Y | Y | N | N |  | Y | Y |  | Y | Y | Y | Y | Y | Y |
| 2 | Sheila F Lumley  2020 | N | N | Y | Y | Y | Y | N | N |  | Y | Y |  | Y | Y | Y | Y | N | N |
| 3 | H. Abo-Leyah  2021 | N | N | Y | Y | Y | Y | N | N |  | Y | Y |  | Y | Y | Y | Y | Y | Y |
| 4 | Anna Jeffery-Smith  2021 | N | N | Y | Y | Y | Y | N | N |  | Y | Y |  | Y | Y | **N^*^** | **Y^*^** | N | N |
| 5 | Raymond A. Harvey  2021 | **Y^*^** | **N^*^** | Y | Y | Y | Y | N | N |  | Y | Y |  | Y | Y | Y | Y | N | N |
| 6 | Adrian M. Shields  2021 | N | N | Y | Y | Y | Y | N | N |  | Y | Y |  | Y | Y | Y | Y | Y | Y |
| 7 | Antonio Leidi  2021 | Y | Y | Y | Y | Y | Y | N | N |  | Y | Y |  | Y | Y | Y | Y | N | N |
| 8 | Candice L. Clarke  2021 | N | N | Y | Y | Y | Y | N | N |  | Y | Y |  | Y | Y | Y | Y | Y | Y |
| 9 | Victoria Jane Hall  2021 | N | N | Y | Y | Y | Y | Y | Y |  | Y | Y |  | Y | Y | Y | Y | Y | Y |
| 10 | Andrew G Letizia  2021 | N | N | Y | Y | Y | Y | Y | Y |  | **N^*^** | **Y^*^** |  | Y | Y | N | N | Y | Y |
| 11 | Mattia Manica  2021 | Y | Y | Y | Y | Y | Y | N | N |  | Y | Y |  | Y | Y | Y | Y | Y | Y |
| 12 | L. J. Abu-Raddad  2021 | **Y^*^** | **N^*^** | **N^#^** | **Y^#^** | Y | Y | N | N |  | Y | Y |  | Y | Y | Y | Y | Y | Y |
| 13 | Maria Krutikov  2021 | N | N | Y | Y | Y | Y | Y | Y |  | **N^#^** | **Y^#^** |  | Y | Y | N | N | N | N |
| 14 | Antonio Leidi  2021 | Y | Y | Y | Y | Y | Y | N | N |  | **Y^*^** | **N^*^** |  | Y | Y | Y | Y | N | N |
| 15 | [Hannah E Maier](https://www.ncbi.nlm.nih.gov/pubmed/?term=Maier%20HE%5bAuthor%5d&cauthor=true&cauthor_uid=34411230)  2021 | Y | Y | Y | Y | Y | Y | Y | Y |  | Y | Y |  | Y | Y | Y | Y | Y | Y |
| 16 | Sebastian Havervall  2021 | N | N | Y | Y | Y | Y | N | N |  | Y | Y |  | Y | Y | N | N | Y | Y |
| 17 | Charles F Schuler 4th  2021 | N | N | Y | Y | Y | Y | N | N |  | Y | Y |  | Y | Y | Y | Y | N | N |
| 18 | Philipp Kohler  2021 | N | N | Y | Y | Y | Y | N | N |  | Y | Y |  | Y | Y | Y | Y | N | N |
| 19 | Luke Muir  2021 | N | N | Y | Y | Y | Y | Y | Y |  | Y | Y |  | Y | Y | Y | Y | Y | Y |
| Consistency (%) | | 89.5 | | 94.7 | | 100.0 | | 100.0 | |  | 84.2 | |  | 100.0 | | 94.7 | | 100.0 | |

N: No; Y: Yes; I1: First investigator; I2: Second investigator;

* The disagreement have been resolved through discussion with third investigator, and the final judgment was “Y”;

# The disagreement have been resolved through discussion with third investigator, and the final judgment was “N”;

^ In the part “Comparability”, “Y” and “N” stand for two stars and one star, respectively.

| Supplementary Table 4. Quality assessment of the included studies using the NOS* | | | | | | | | | | | | | |
| --- | --- | --- | --- | --- | --- | --- | --- | --- | --- | --- | --- | --- | --- |
| NO. | Study | Selection | | | |  | Comparability |  | Outcome | | |  | Scores^#^ |
|  |  | Representativeness of the exposed cohort | Selection of the non-exposed cohort | Ascertainment of exposure | Demonstration that outcome of interest was not present at start of study |  | Comparability of cohorts on the basis of the design or analysis |  | Assessment of outcome | Was follow up long enough for outcomes to occur | Adequacy of follow up of cohorts |  |  |
| 1 | John T. Wilkins MD  et al, 2020 |  |  |  |  |  |  |  |  |  |  |  | 7 (HQ) |
| 2 | Sheila F Lumley  et al, 2020 |  |  |  |  |  |  |  |  |  |  |  | 6 (MQ) |
| 3 | H. Abo-Leyah  et al, 2021 |  |  |  |  |  |  |  |  |  |  |  | 6 (MQ) |
| 4 | Anna Jeffery-Smith  et al, 2021 |  |  |  |  |  |  |  |  |  |  |  | 5 (MQ) |
| 5 | Raymond A. Harvey  et al, 2021 |  |  |  |  |  |  |  |  |  |  |  | 6 (MQ) |
| 6 | Adrian M. Shields  et al, 2021 |  |  |  |  |  |  |  |  |  |  |  | 7 (HQ) |
| 7 | Antonio Leidi  et al, 2021 |  |  |  |  |  |  |  |  |  |  |  | 7 (HQ) |
| 8 | Candice L. Clarke  et al, 2021 |  |  |  |  |  |  |  |  |  |  |  | 6 (MQ) |
| 9 | Victoria Jane Hall  et al, 2021 |  |  |  |  |  |  |  |  |  |  |  | 8 (HQ) |
| 10 | Andrew G Letizia  et al, 2021 |  |  |  |  |  |  |  |  |  |  |  | 7 (HQ) |
| 11 | Mattia Manica  et al, 2021 |  |  |  |  |  |  |  |  |  |  |  | 8 (HQ) |
| 12 | L. J. Abu-Raddad  et al, 2021 |  |  |  |  |  |  |  |  |  |  |  | 6 (MQ) |
| 13 | Maria Krutikov  et al, 2021 |  |  |  |  |  |  |  |  |  |  |  | 5 (MQ) |
| 14 | Antonio Leidi  et al, 2021 |  |  |  |  |  |  |  |  |  |  |  | 7 (HQ) |
| 15 | [Hannah E Maier](https://www.ncbi.nlm.nih.gov/pubmed/?term=Maier%20HE%5bAuthor%5d&cauthor=true&cauthor_uid=34411230)  et al, 2021 |  |  |  |  |  |  |  |  |  |  |  | 8 (HQ) |
| 16 | Sebastian Havervall  et al, 2021 |  |  |  |  |  |  |  |  |  |  |  | 5 (MQ) |
| 17 | Charles F Schuler 4th  et al, 2021 |  |  |  |  |  |  |  |  |  |  |  | 5 (MQ) |
| 18 | Philipp Kohler  2021 |  |  |  |  |  |  |  |  |  |  |  | 5 (MQ) |
| 19 | Luke Muir  2021 |  |  |  |  |  |  |  |  |  |  |  | 7 (HQ) |

LQ: Low quality; MQ: Moderate quality; HQ: High quality;

* The empty star indicates this study did not get any scores for that category.

^#^ A score of 0–3 solid stars was considered as a LQ study, a score of 4–6 solid stars was considered as a MQ study, and a score of 7–9 solid stars was considered as a HQ study.

# Supplementary Figure 1. Sensitivity analysis for pooled incidence rate ratio


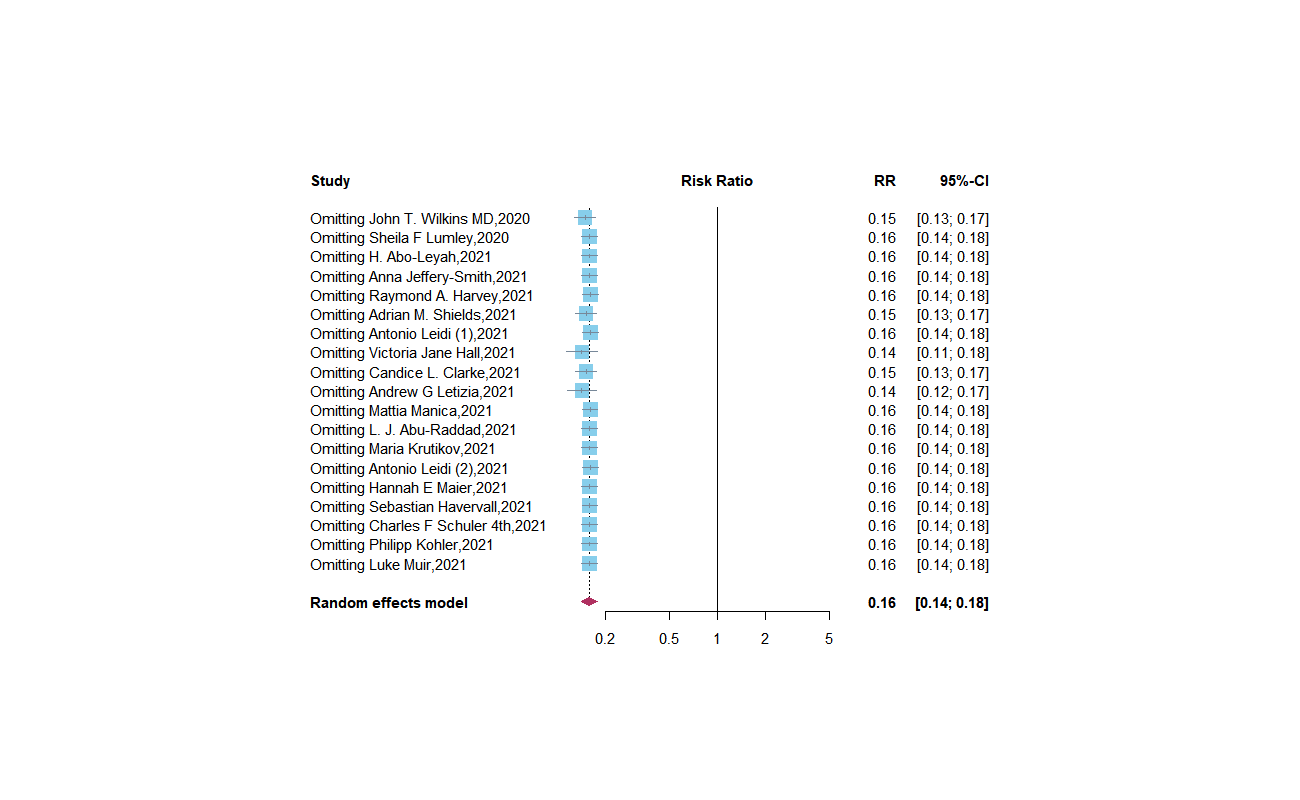


# Supplementary Figure 2. Funnel plot for publication bias


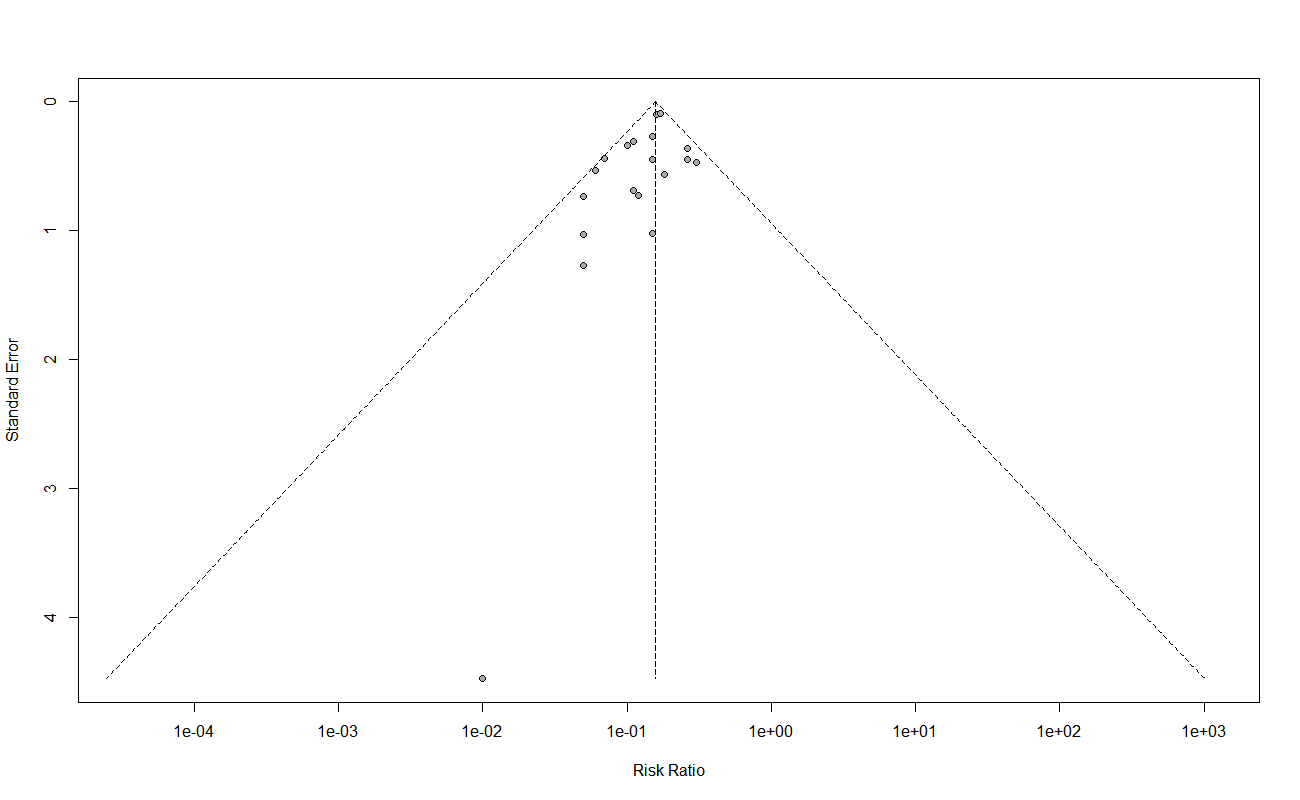


# Supplementary Figure 3. Forest plot of the pooled incidence rate ratio for SARS-CoV-2 infection comparing baseline seropositive with seronegative individuals in HCWs subgroup and general population subgroup


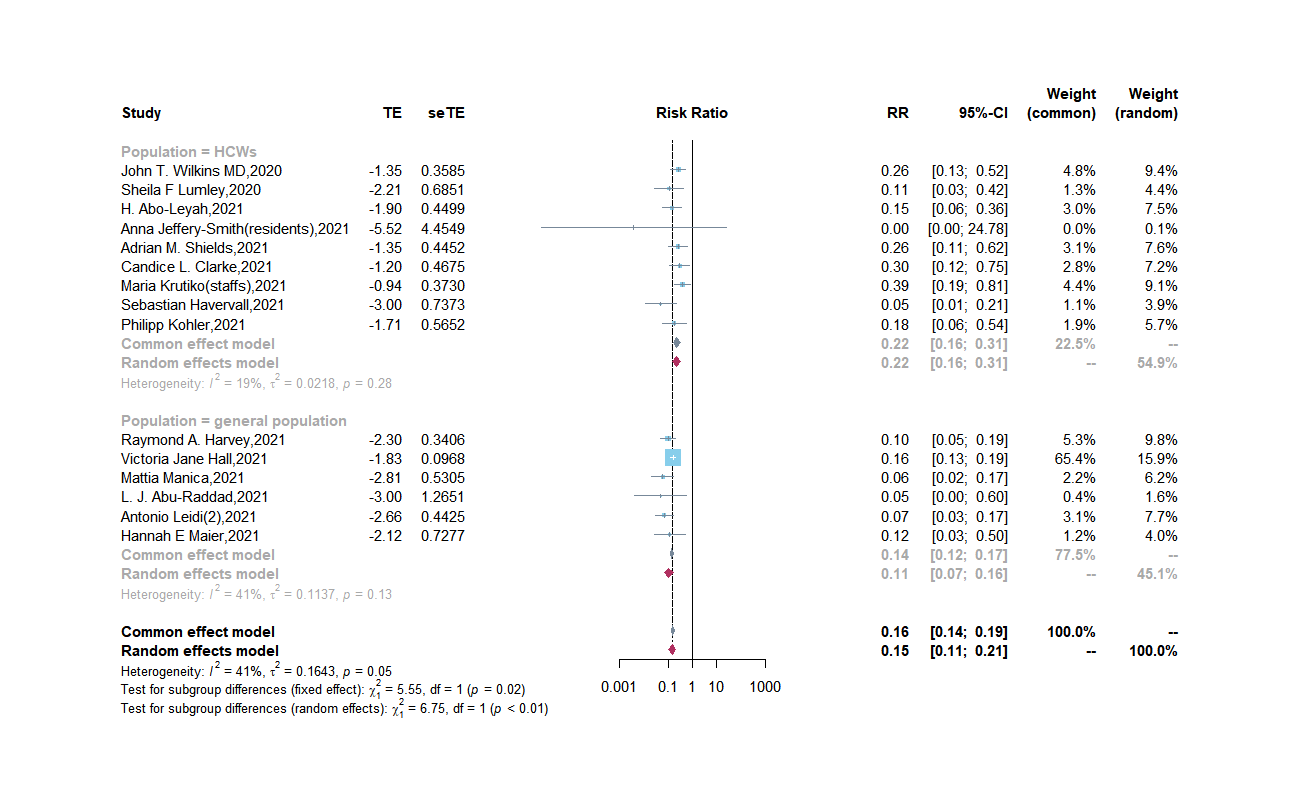


# Supplementary Figure 4. Forest plot of the pooled incidence rate ratio for SARS-CoV-2 infection comparing baseline seropositive with seronegative individuals in <60 years old subgroup and ≥60 years old subgroup


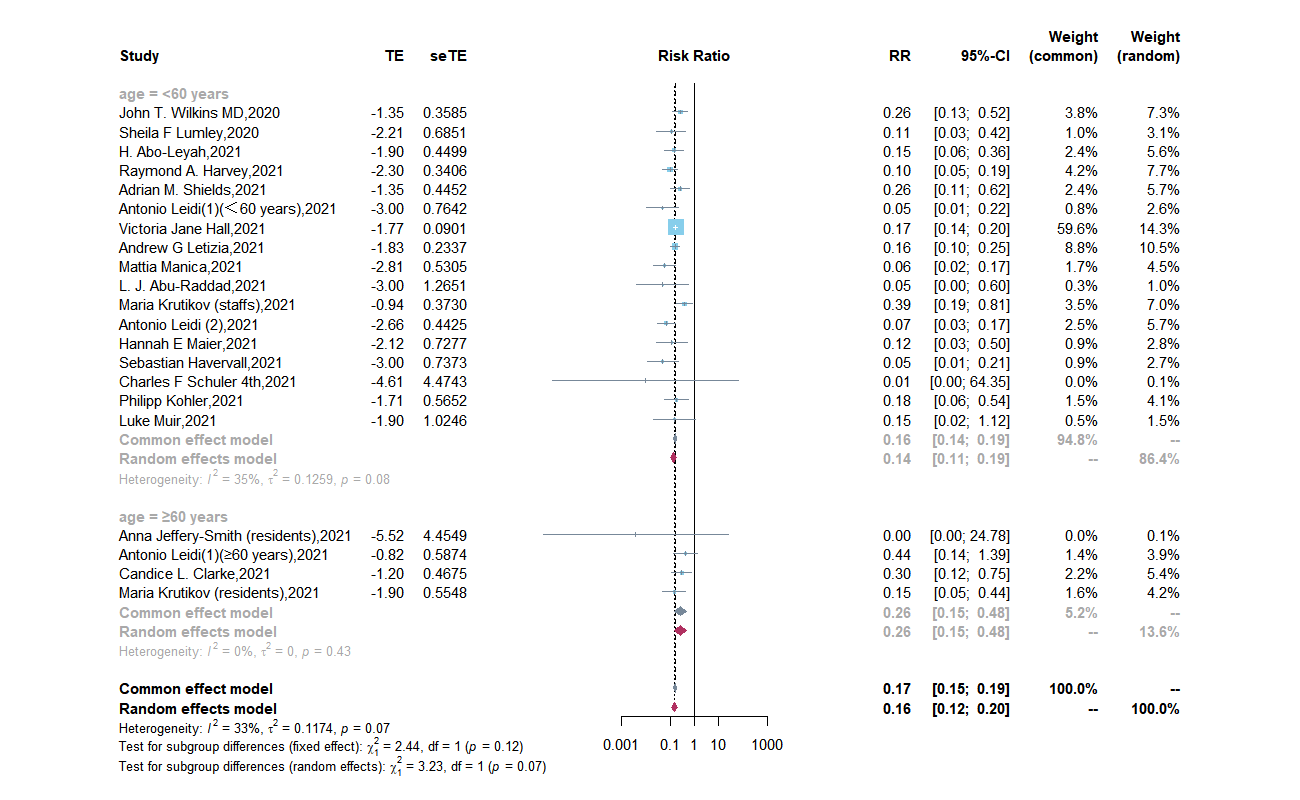


# Supplementary Figure 5. Forest plot of the pooled incidence rate ratio for SARS-CoV-2 infection comparing baseline seropositive with seronegative individuals in peer-review subgroup and preprint subgroup


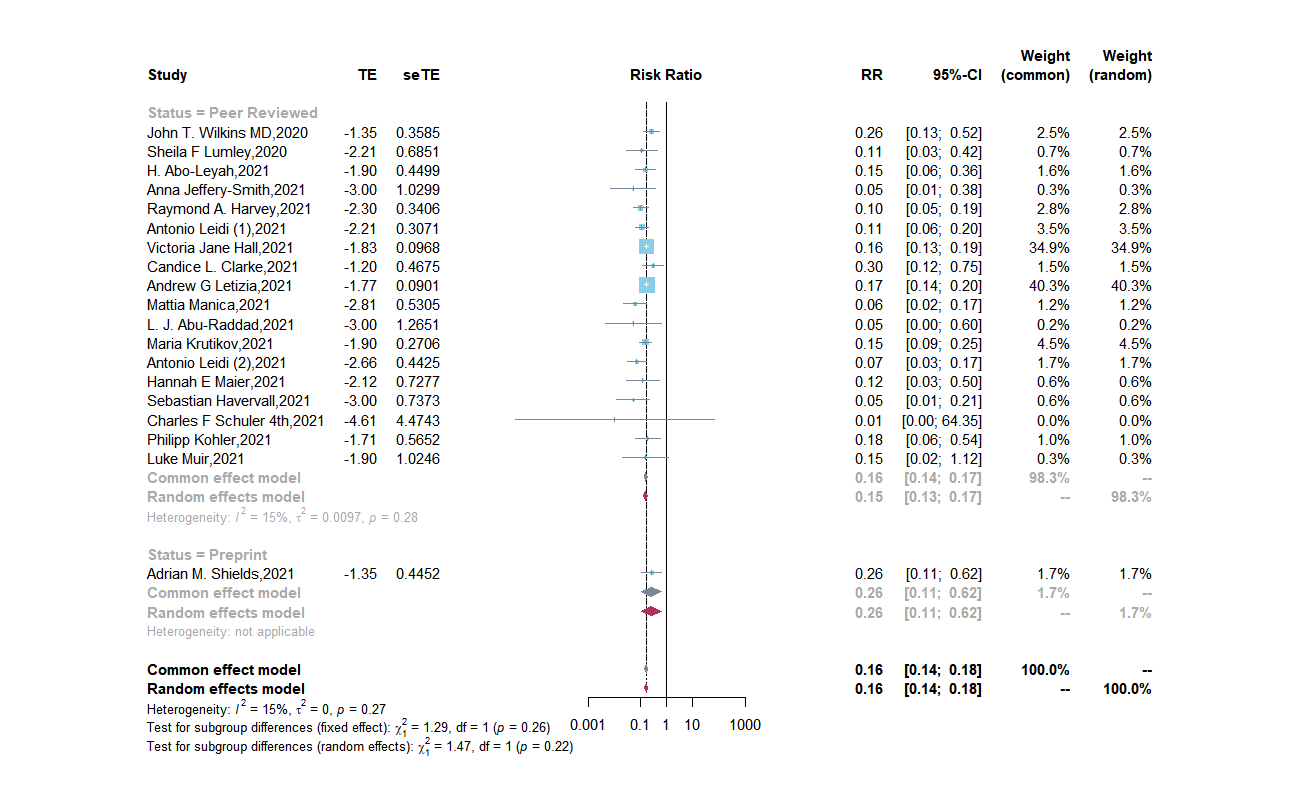


# Supplementary Figure 6. Forest plot of the pooled incidence rate ratio for SARS-CoV-2 infection comparing baseline seropositive with seronegative individuals in S protein subgroup and N protein subgroup


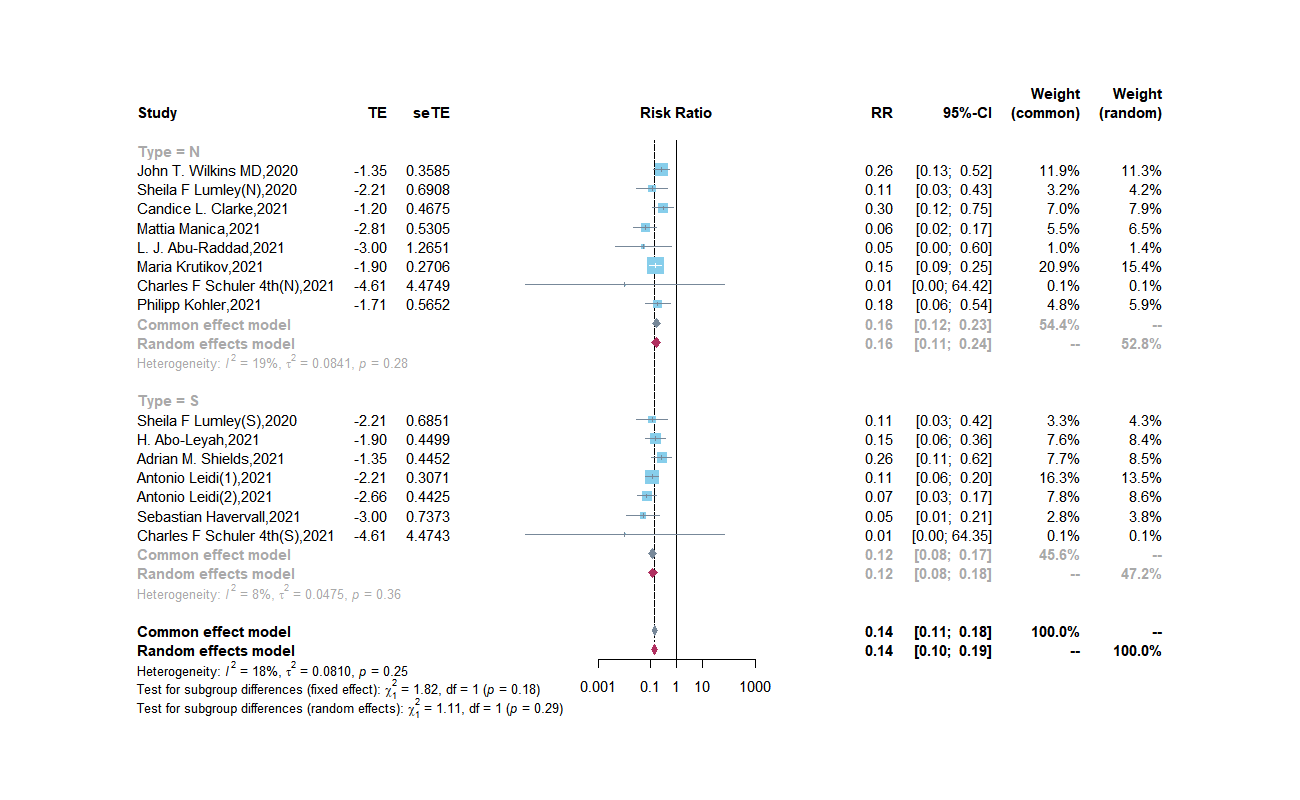


# Supplementary Figure 7. Forest plot of the pooled incidence rate ratio for SARS-CoV-2 infection comparing baseline seropositive with seronegative individuals in adjusted subgroup and unadjusted subgroup


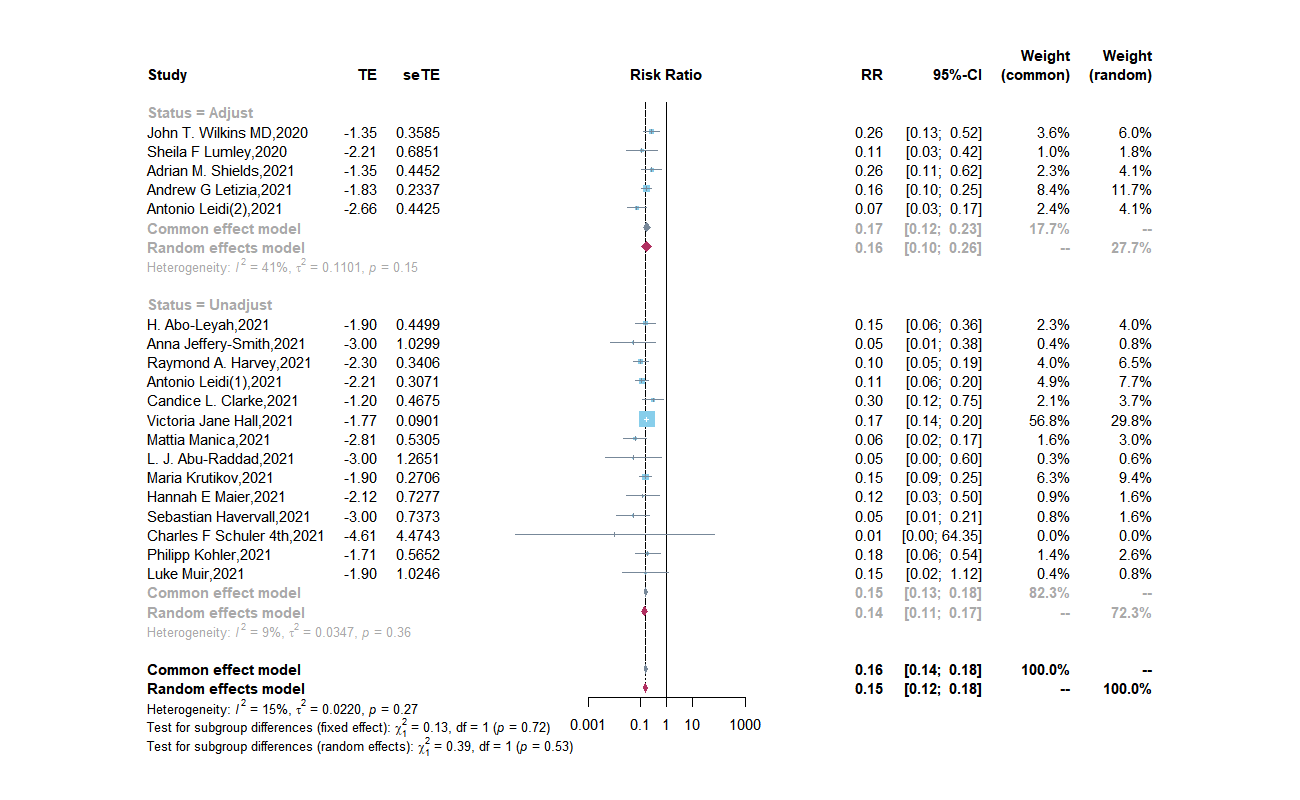


# Supplementary Figure 8. Forest plot of the pooled incidence rate ratio for SARS-CoV-2 infection comparing baseline seropositive with seronegative individuals in passive monitoring subgroup and active monitoring subgroup


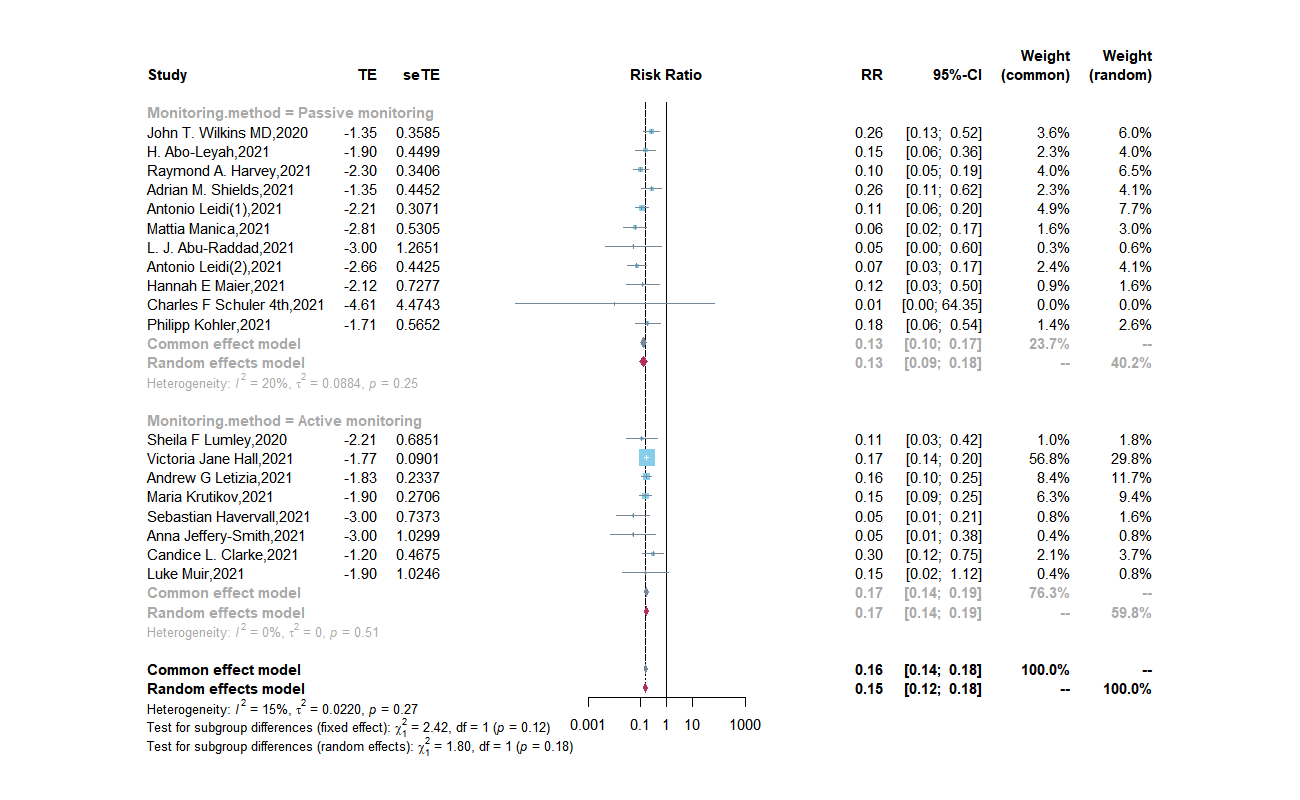


# Supplementary Figure 9. Forest plot of the pooled incidence rate ratio for SARS-CoV-2 infection comparing baseline seropositive with seronegative individuals in strict definition subgroup and loose definition subgroup


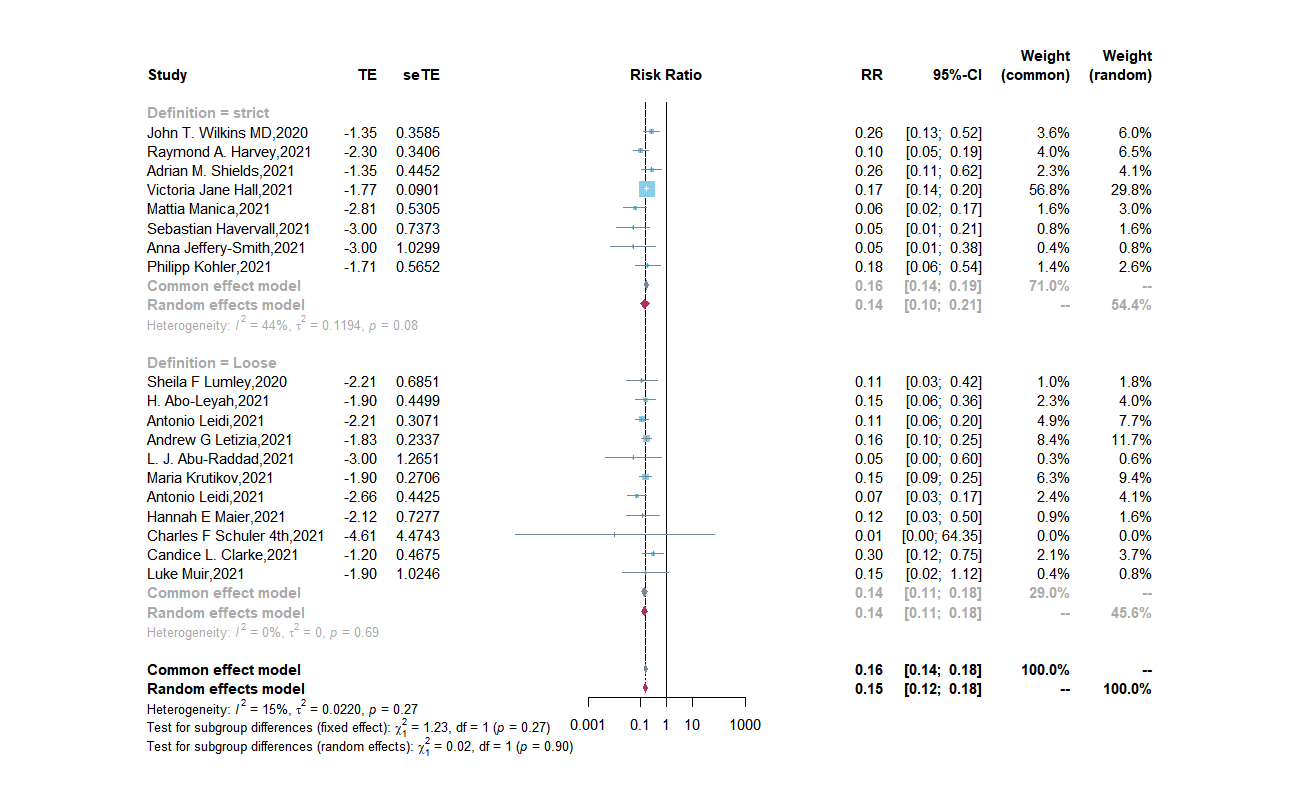


# Supplementary Figure 10. The changing trend of incidence rate ratio after prior infection


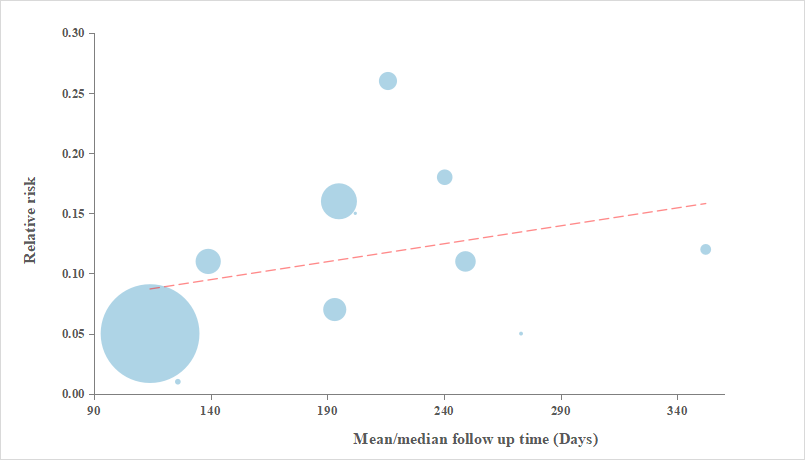

Supplement: Supplemental Material [file TEMI_A_2046446_SM4746.docx]
